# Cooperative binding of the tandem WW domains of PLEKHA7 to PDZD11 promotes conformation-dependent interaction with tetraspanin 33

**DOI:** 10.1074/jbc.RA120.012987

**Published:** 2020-05-05

**Authors:** Florian Rouaud, Francesca Tessaro, Laura Aimaretti, Leonardo Scapozza, Sandra Citi

**Affiliations:** 1 Department of Cell Biology, Faculty of Sciences, University of Geneva, Geneva, Switzerland; 2 The Institute for Genetics and Genomics in Geneva (iGE3), University of Geneva, Geneva, Switzerland; 3 The Pharmaceutical Biochemistry Group, School of Pharmaceutical Sciences, University of Geneva, Geneva, Switzerland

**Keywords:** Pleckstrin homology domain containing A7 (PLEKHA7), PDZ domain containing 11 (PDZD11), tetraspanin 33, WW domain, polyproline, cooperativity, cell-cell junction, α-toxin, ADAM metallopeptidase domain 10 (ADAM10), adherens junction, membrane protein, molecular docking, protein complex, PDZD11, PLEKHA7, polyproline, tetraspanin33

## Abstract

Pleckstrin homology domain–containing A7 (PLEKHA7) is a cytoplasmic protein at adherens junctions that has been implicated in hypertension, glaucoma, and responses to *Staphylococcus aureus* α-toxin. Complex formation between PLEKHA7, PDZ domain–containing 11 (PDZD11), tetraspanin 33, and the α-toxin receptor ADAM metallopeptidase domain 10 (ADAM10) promotes junctional clustering of ADAM10 and α-toxin–mediated pore formation. However, how the N-terminal region of PDZD11 interacts with the N-terminal tandem WW domains of PLEKHA7 and how this interaction promotes tetraspanin 33 binding to the WW1 domain is unclear. Here, we used site-directed mutagenesis, glutathione *S*-transferase pulldown experiments, immunofluorescence, molecular modeling, and docking experiments to characterize the mechanisms driving these interactions. We found that Asp-30 of WW1 and His-75 of WW2 interact through a hydrogen bond and, together with Thr-35 of WW1, form a binding pocket that accommodates a polyproline stretch within the N-terminal PDZD11 region. By strengthening the interactions of the ternary complex, the WW2 domain stabilized the WW1 domain and cooperatively promoted the interaction with PDZD11. Modeling results indicated that, in turn, PDZD11 binding induces a conformational rearrangement, which strengthens the ternary complex, and contributes to enlarging a “hydrophobic hot spot” region on the WW1 domain. The last two lipophilic residues of tetraspanin 33, Trp-283 and Tyr-282, were required for its interaction with PLEKHA7. Docking of the tetraspanin 33 C terminus revealed that it fits into the hydrophobic hot spot region of the accessible surface of WW1. We conclude that communication between the two tandem WW domains of PLEKHA7 and the PLEKHA7–PDZD11 interaction modulate the ligand-binding properties of PLEKHA7.

Cell-cell junctions are implicated in developmental, physiological, and pathological processes. PLEKHA7, a protein localized at cadherin-based adherens junctions, was originally discovered as an interactor of p120-catenin (1) and paracingulin (2, 3), and its interaction with microtubules, through the minus-end binding protein nezha/CAMSAP3, stabilizes cadherin-based junctions and the barrier function of epithelia (1, 4). At the tissue and organism level, PLEKHA7 has been implicated in morphogenesis and disease. Genome-wide association studies show that single nucleotide polymorphism in the PLEKHA7 gene are associated with human hypertension and high systolic pressure (5), and with primary angle closure glaucoma (6). In agreement, rats KO for PLEKHA7 show an attenuated increase in blood pressure and kidney damage induced by a high salt diet, and increased intracellular calcium and nitrous oxide signaling in endothelial cells (7). In a zebrafish model, the PLEKHA7 homolog Hadp1 is required for cardiac morphogenesis and contractility (8). PLEKHA7 has also been involved in the control of microRNA processing (9, 10) and Rho GTPase activity (11). In cultured cells and mice, PLEKHA7 is required to make cells more susceptible to the cytotoxic effects of *Staphylococcus aureus* α-toxin, through a mechanism that involves junctional clustering of ADAM10, mediated by tetraspanin 33 (Tspan33) (12, 13).

Considering the important roles of PLEKHA7 in physiology and disease, it is crucial to understand the molecular, cellular, and structural basis for its functions. PLEKHA7 comprises N-terminal tandem WW, pleckstrin homology, and coiled-coil domains, and interacts with different junctional proteins (1, 3, 14) and membrane phospholipids (8). We identified PDZD11 as the highest affinity interactor of PLEKHA7 using 2-hybrid screens and proteomic approaches, and showed that the interaction of the N-terminal region of PDZD11 with the WW1 domain of PLEKHA7 is essential to cluster the Ig-like adhesion molecules nectins at adherens junctions, and promote efficient junction assembly (15). The same interaction is also required to dock the *Staphylococcus aureus* α-toxin receptor ADAM10 at junctions, through binding of the WW1 domain of PLEKHA7 to the ADAM10 chaperone Tspan33 (13). Thus, the interaction with PDZD11 is critical in mediating the activity of PLEKHA7 as a scaffold for transmembrane proteins, such as nectins and Tspan33. However, nothing is known about the structural basis for the interaction of PLEKHA7 with PDZD11, and the mechanisms through which PDZD11 promotes PLEKHA7 binding to Tspan33.

WW domains are domains of 35-40 amino acids, characterized by two highly conserved Trp residues, separated by 20 to 23 amino acids. WW domains fold into a very stable structure with three antiparallel β-sheets, and can accommodate a high degree of sequence variability (16, 17). They typically bind either to proline-rich sequences or phosphorylated Ser or Thr residues, and are found in many human proteins, which play important roles in nuclear signaling, protein stabilization, the assembly of multiprotein networks, and diseases (18–20). Because of the critical importance of the PDZD11 interaction in PLEKHA7 function, we sought to gain further insights into the structural aspects of the interaction between the WW domains of PLEKHA7 and PDZD11, using site-directed mutagenesis, GST (glutathione-S-transferase) pulldown assays, and immunofluorescence. Our data allows to generate a model for the structure of the complex using molecular modeling and docking studies, which provides a rational mechanism through which intramolecular WW1–WW2 interactions promote binding to PDZD11, and this in turn promotes interaction with Tspan33.

## Results

### Specific mutations within the WW1 domain of PLEKHA7 reduce binding to PDZD11, but the WW2 domain rescues the effects of these mutations

**Figure 1.**
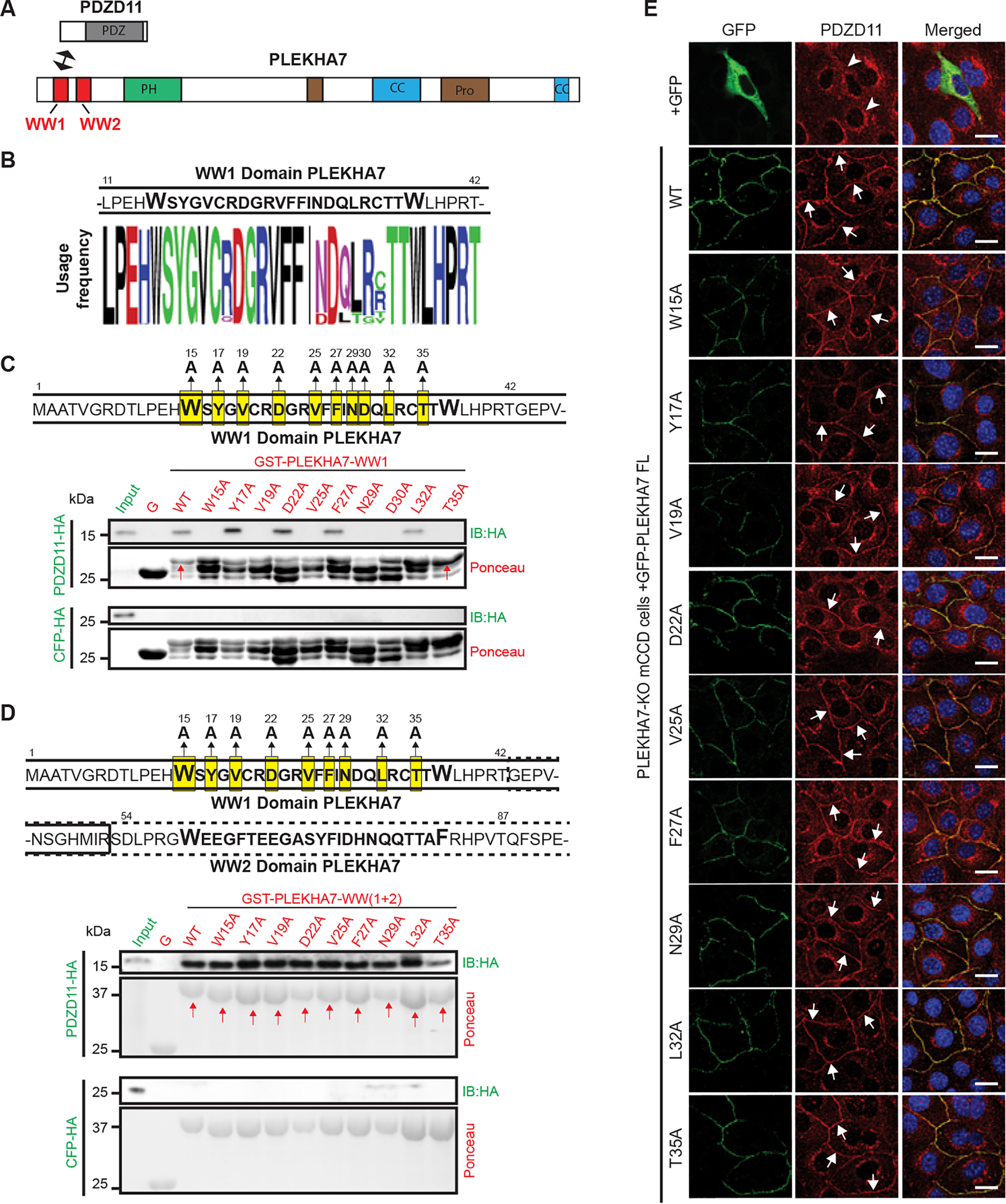
**The WW2 domain of PLEKHA7 stabilizes the WW1 domain and rescues the interaction of WW1 mutants with PDZD11.** *A,* schematic diagrams of PDZD11 (*top*) and PLEKHA7 (*bottom*), with the indicated structural domains. The N-terminal sequence of PDZD11 (P7-ID: PLEKHA7-interaction domain) interacts with the WW1 domain of PLEKHA7 (*arrow*) (15). *B, top*: sequence of the WW1 domain of PLEKHA7. *Bottom*: Weblogo diagram of residue conservation. *C, top*: sequence of WW1 domain, with mutations of highly conserved residues (to Ala) highlighted in *yellow*. The *numbers above* each residue indicate residue number in the sequence. *Bottom*: immunoblot analysis of GST pulldowns using GST (*G*) fused to WT and mutant WW1 domains as bait, and either PDZD11-HA or CFP-HA as preys. GST and GST fusion baits are indicated in *red*, and preys are indicated in *green*. Ponceau S-stained blots below immunoblots show baits. *Red arrows* indicate baits showing no or little proteolytic degradation. *D, top*: sequence of WW1 (*continuous line*, residues 1–53) and WW2 (*dotted line*, residues 43–98) domains, with highlighted WW1 mutations. The sequence linking the WW1 to the WW2 domain is in a *dotted box*. *Bottom,* immunoblot analysis of GST pulldowns using GST fused to WT and mutant WW1 + WW2 domains as bait, and either PDZD11-HA or CFP-HA as preys. Ponceau S-stained blots *below* the immunoblots show baits. *E,* immunofluorescence localization of endogenous PDZD11 in PLEKHA7-KO mCCD cells rescued either with GFP, or with either WT or WW1 point mutants of GFP-tagged full-length PLEKHA7. *Merged* images show nuclei in *blue* (DAPI). *Arrows* indicate junctional labeling. *Arrowheads* indicate decreased/undetected junctional labeling. *Bar* = 20 μm.

The N-terminal region of PDZD11 interacts with the WW1, but not the WW2 domain of PLEKHA7 (Fig. 1*A*) (15). However, the interaction is stronger when both WW1 and WW2 domains are present (13, 15). The sequences of the WW1 (Fig. 1*B*) and WW2 (Fig. S1*A*) domains are highly conserved, because >90% of residues are identical between different species (26/32 in WW1, 24/26 in WW2). To identify the residues involved in the WW1-PDZD11 interaction and the mechanism through which the WW2 domain promotes binding of PDZD11 to PLEKHA7 we used site-directed mutagenesis. We generated alanine mutants within the WW1 domain at amino acid positions 15, 17, 19, 22, 25, 27, 29, 30, 32, and 35 (Fig. 1*C*). The WT and mutant WW1 sequences were fused downstream of GST to generate baits for pulldown assays, where the preys were either PDZD11-HA or CFP-HA (negative control) (Fig. 1*C*). Immunoblot analysis showed that the WT sequence and the mutants Y17A, D22A, F27A, and L32A interacted with PDZD11, whereas the W15A, V19A, V25A, N29A, D30A, and T35A mutants failed to detectably interact with PDZD11 (Fig. 1*C*, *top*). None of the baits interacted with negative control HA-tagged CFP (Fig. 1*C*, *bottom*). Ponceau S staining of the blots revealed that all the WW1 mutant constructs migrated as multiple bands, typically three polypeptides, with different relative intensities (Fig. 1*C*, *Ponceau*), suggesting that the GST fusion proteins are unstable, and subjected to partial proteolytic degradation. Among mutants that failed to interact with PDZD11, T35A showed the least degree of degradation and migrated similar to WT, as indicated by the prevalence of the higher molecular size polypeptide (Fig. 1*C*, *red arrows*). Next, we asked whether mutations of the WW1 domain that resulted in decreased interaction with PDZD11 were also effective in the context of constructs comprising both WW1 and WW2 domains. Thus, we generated fusion proteins comprising the first 160 residues of PLEKHA7 (*e.g.* both WW1 and WW2 domains and a short downstream sequence, but not the pleckstrin homology domain, Fig. 1*A*), either with WT sequence, or with the single amino acid mutations within the WW1 domain (Fig. 1*D*). Immunoblot analysis showed that all mutant baits interacted with PDZD11 as efficiently as WT, and none with CFP-HA (Fig. 1*D*). In addition, Ponceau S staining of GST fusion proteins showed no degradation, because all fusion proteins migrated as one major polypeptide (Fig. 1*D*, *Ponceau, red arrows*). These results suggest that the presence of the WW2 domain stabilizes the WW1 domain. Next, to obtain separate evidence that these results reflect physiologically relevant mechanisms and interactions, we examined the effect of WW1 mutations on the interaction between full-length PLEKHA7 and PDZD11 in cells. PLEKHA7 recruits PDZD11 to junctions (15), and we used as a readout the rescue of the junctional localization of PDZD11, which is abolished in PLEKHA7-KO cells, by different WW domain mutants of PLEKHA7. In PLEKHA7-KO cells transfected with GFP alone, no PDZD11 labeling was detected at junctions (negative control, Fig. 1*E*, +*GFP, arrowheads*). In contrast, endogenous PDZD11 was rescued to junctions when PLEKHA7-KO cells where transfected with GFP-tagged full-length WT PLEKHA7 (Fig. 1*E*, *WT*, *arrows*) (15). Consistent with the GST pulldown results carried out for the WW(1 + 2) domains, all the full-length PLEKHA7 constructs harboring WW1 domain mutations rescued the junctional localization of endogenous PDZD11 (Fig. 1*E*). Yet, deletion of the WW1 domain prevented the rescue of the junctional localization of PDZD11, confirming the critical role of the WW1 domain in PLEKHA7 interaction with PDZD11 (Fig. S1*B*). In summary, these results identify residues in the WW1 domain that are required for the interaction with PDZD11 by GST pulldown, suggesting that they are either directly implicated in the interaction, or they are required to maintain the correct folding of the WW1 domain. Moreover, because the presence of the WW2 domain offsets the effects of specific mutations on the interaction between PLEKHA7-PDZD11 both by GST pulldown and in cells, we conclude that the WW2 domain protects the WW1 domain from the de-stabilizing effects of the WW1 mutations. Therefore, we next wondered whether mutations within the WW2 domain might affect the ability of the WW1 domain to bind to PDZD11. Thus, we generated mutations within the WW2 domain, at positions 59, 60, 61, 65, 68, 71, 73, 75, 76, 84, and 85 (Fig. S1*C*, *top*). None of the mutations affected the ability of the WW1 + WW2 domain to interact with PDZD11, either by GST pulldown (Fig. S1*C*, *bottom*) or in cells (Fig. S1*D*). These results indicate that the structure of the WW1 domain and its ability to interact with PDZD11 is resistant to several mutations within the conserved residues of the WW2 domain.

### His-75 of the WW2 domain cooperates with Asp-30 and/or Thr-35 of the WW1 domain of PLEKHA7 to promote interaction with PDZD11

**Figure 2.**
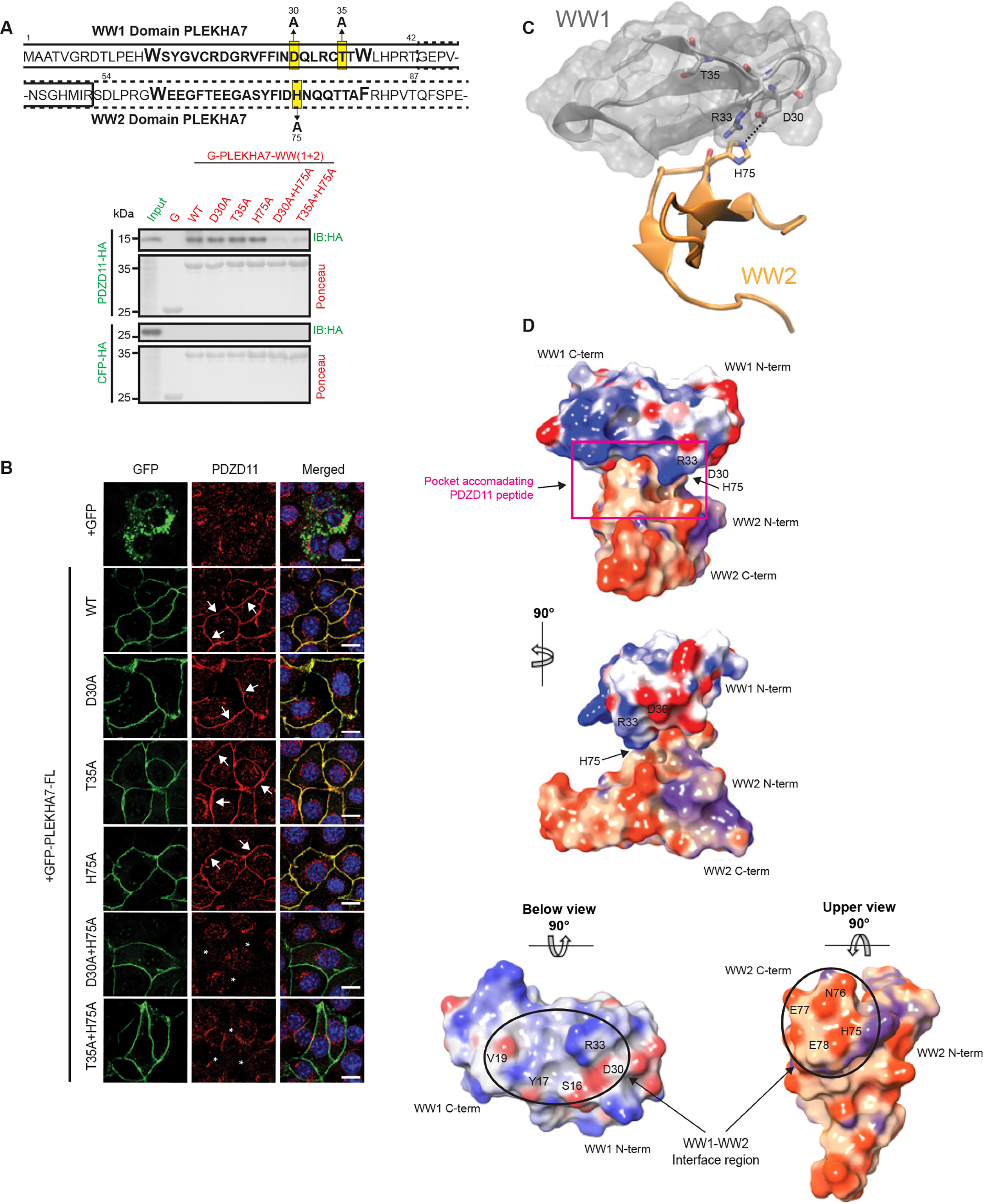
**Binding of PDZD11 to the cleft between WW1 and WW2 domains requires either Asp-30 or Thr-35 of WW1 and His-75 of WW2.** *A, top*: sequence of WW1 + WW2 domains, with mutations in residues within WW1 and WW2 highlighted in *yellow*. The *numbers above* and *below* each residue indicate residue number in the sequence. *Bottom*: immunoblot analysis of GST pulldowns using GST (*G*) fused to WT and mutant WW1 + WW2 domains as bait, and either PDZD11-HA or CFP-HA as preys. *B,* immunofluorescence localization of endogenous PDZD11 in PLEKHA7-KO mCCD cells rescued with either GFP, or with either WT or WW1 point mutants or WW2 point mutants, or WW1 + WW2 point mutants of GFP-tagged full-length PLEKHA7. *Merged* images show nuclei in *blue* (DAPI). *Arrows* indicate junctional labeling and *asterisks* indicate reduced/undetectable labeling. *Bar* = 20 μm. *C,* model of interaction between the WW1 domain (*gray*, with surface representation) and WW2 domain (*orange*). The *dotted black line* shows the hydrogen bond occurring between His-75 and Asp-30. *D,* electrostatic potential surface (EPS) of the WW1 and WW2 domains. The color ramp is set with a minimum value of −0.2 (*red*) and a maximum of 0.2 (*blue*). The WW2 domain presents in transparency an *orange* molecular surface for the EPS = 0 instead of the *white* used for WW1 to help in distinguish of the two domains. The probe radius to define the accessible surface area was set to 1.4 Å. The image shows the molecule from different space orientations turning along the *y* and *x* axes. In the *upper part* of the panel, a *violet rectangle* indicates the pocket that accommodate PDZD11. In the *panel below* the interacting surface between the two domains is shown from the below (+ 90°) and upper (−90°) views, respectively. The WW2 and WW1 domains are not displayed to allow the visualization of the contact surface indicated by the *black circle*. Images were obtained using the Molecular Surface tool from Maestro (Schrödinger release 2016-4) program.

Next, we sought to clarify the mechanism of WW1 stabilization by WW2, by attempting to find combinations of mutations within the two domains that would affect the interaction with PDZD11. We selected two mutations within the WW1 domain: the T35A mutation, which showed the least degradation of the recombinant bait protein (Fig. 1*C*), or the nearby D30A mutation, which also showed strongly decreased interaction with PDZD11 (Fig. 1*C*). When we combined either the T35A or D30A mutations in the WW1 domain with different mutations within the WW2 domain, only T35A + H75A and D30A + H75A combinations resulted in the complete loss of binding to the PDZD11 prey by GST pulldown assay (Fig. 2*A* and Fig. S2, *A* and *C*), and in a failure to rescue the junctional localization of PDZD11 within cells (Fig. 2*B*). Other combinations of mutations could rescue junctional PDZD11 (Fig. S2*B*), demonstrating that the results of GST pulldowns reflect physiologically relevant interactions and are not artifacts. Finally, when combining together either the D30A or T35A mutations with mutations of the residues flanking His-75 within the WW2 domain, *e.g.* either D74A or N76A, the interaction with PDZD11 was maintained (Fig. S2, *C–E*). These results were confirmed when we switched bait and prey, *e.g.* we used GST-PDZD11 as a bait, and either WT or mutant constructs of the WW1 + WW2 domains of PLEKHA7 as preys. The single mutations D30A, T35A, and H75A did not affect PDZD11 interaction with the WW1 + WW2 domains (Fig. S2*G*), but the double mutants, either D30A + H75A, or T35A+H75A resulted in loss of interaction with PDZD11 (Fig. S2*H*).

Next, we combined the information derived from these mutagenesis studies with structural modeling to gain further understanding on the structural basis of the PLEKHA7-PDZD11 interaction. Based on the homology of the WW1 and WW2 domains with templates of the ubiquitin ligase Nedd4 (48% sequence identity with WW1) and the Syntaxin-binding protein (58% sequence identity with WW2), we modeled a three antiparallel β-sheet structures of the WW1 and WW2 domains (Fig. S3, *A* and *B*) (22). As a validation of the correct geometry of the models, the amino acid residues were located in the “allowed” regions of the Ramachandran plot (Fig. S3, *C* and *D*). Based on the models and on the experimental results highlighting the importance of the His-75 of the WW2 domain together with Asp-30 and Thr-35 of the WW1 domain, we carried out a series of molecular docking analyses, which allowed to model the interaction between the WW1 and WW2 domains (Fig. 2, *C* and *D*). According to this model, His-75 of WW2 is accommodated in a small pocket of the WW1 domain formed by residues Asp-30, Arg-33, Tyr-17, and Ser-16 (Fig. 2*D*, see below). Although the contact surface between the two domains is small (549 Å^2^) compared to the overall accessible surface area of the complex (5546.75 Å^2^), the electrostatic potential surface at the interface of the two domains shows good complementarity (Fig. 2*D*). Residues Asn-76, Glu-77, and Glu-78, at the interface of WW2 domain, electrostatically contribute to strengthening the interaction (Fig. 2*D*, see below). In addition, His-75 forms a hydrogen bond with Asp-30 of WW1, thus generating a “binding pocket” suitable for accommodating PDZD11 at the interface between the two domains (Fig. 2*D*, *red rectangle*). The specific role for His-75 was highlighted by the observation that mutations in neighboring amino acids (D74A, N76A) had no effects on the interaction with PDZD11, either alone or in combination with either D30A or T35A (Fig. S2, *C*–*F*). In agreement, structural inspection of the models reveals that Asp-74 and Asn-76 face away from WW1 domain, suggesting that they are not involved in the interaction between the two domains. Because in our model Arg-33 is localized in the binding pocket close to Asp-30 (Fig. 2*C*), we tested its relevance by combining the mutation R33A with mutations of D74A, H75A, or N76A. These double mutants interacted well with PDZD11 by GST pulldown and in cells (Fig. S2, *E* and *F*), indicating that His-75 of WW2 is the main interactor of Asp-30 of WW1 (Fig. 2*C*).

**Figure 3.**
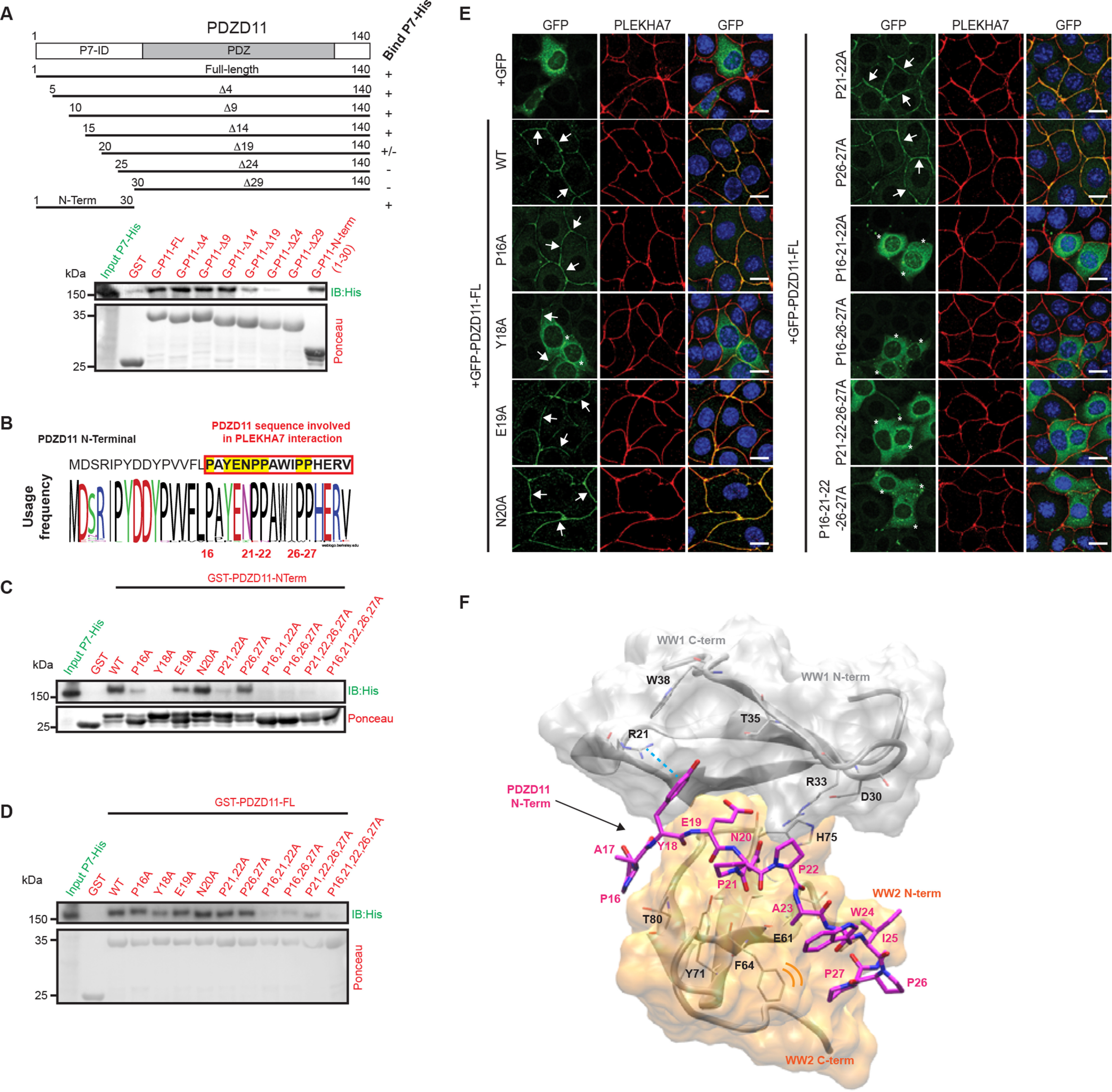
**Polyproline stretches and tyrosine 18 in the N-terminal region of PDZD11 are required for its interaction with PLEKHA7.** *A, top*: schematic domain organization of PDZD11, and schemes of truncated constructs used in GST pulldown assays, and their interaction (Bind P7-His) with full-length PLEKHA7 (indicated by +, ±, −). *Bottom*: immunoblot analysis of GST pulldowns using either GST (*G*), or GST fused to either full-length PDZD11 or the truncated constructs (shown on *top*) as baits, and full-length PLEKHA7-His as prey. Ponceau S-stained blots *below* the immunoblots show baits. *B,* sequence of the P7-ID domain (15) of PDZD11, and Weblogo diagram of residue conservation within this region. *C,* immunoblot analysis of GST pulldowns using GST (*G*) fused to either WT or mutated P7-ID N-terminal domain of PDZD11 as bait and PLEKHA7-His as prey. *D,* immunoblot analysis of GST pulldowns using either GST, or GST fused to either WT or mutated full-length PDZD11 as bait, and PLEKHA7-His as prey. Ponceau S-stained blots *below* immunoblots show baits. *E,* immunofluorescence localization of endogenous PLEKHA7 in WT mCCD cells, transiently transfected with either GFP, or WT or mutants GFP-tagged full-length PDZD11. *Merged* images show nuclei in *blue* (DAPI). *Arrows* indicate junctional labeling, and *asterisks* indicate decreased/undetectable junctional labeling. *Bar* = 20 μm. *F,* binding mode of the PDZD11 dodecapeptide (PAYENPPAWIPP) in the pocket formed by WW1–WW2 domains. PDZD11 is shown in *magenta* as licorice style, whereas the WW1 and WW2 domains are represented, respectively, in *gray* and *orange* as a new cartoon and transparent surface style. The *cyan dotted line* indicates the cation-π interaction of Tyr-18 with Arg-21 in the WW1 domain. Hydrophobic contacts in the WW2 domain are represented with *orange curved lines* (near Phe-64), which indicate the pocket accommodating the tandem prolines 26-27. The image was generated with VMD software.

The WW1–WW2 complex resulted to be stable in time during the 120 ns of molecular dynamic (MD) simulations, as shown by the low values of root mean square deviation (RMSD) (Table S1), specifically in the last 60 ns of the trajectory (Fig. S3*E*, *WW(1* + *2)-WT*). The maintenance of a close distance between the two domains is shown also by the clustered structures from MD simulation, showing the representative conformations assumed by the WW(1 + 2)-WT complex along the simulating time (Fig. S3*G*). In agreement, hydrogen bond frequencies revealed that the main interacting residues were localized at the interface between the two domains, which concurred at the maintenance of the complex stability (Table S2). Importantly, molecular dynamic simulations also showed that the WW1–WW2 system carrying the double point mutations T35A+H75A and D30A+H75A appear more unstable in terms of RMSD values compared either to the WT system or to single mutants D30A and T35A (Fig. S3*E* and Table S1). As was expected by analyzing the overall complex, we noticed that the residues located at the N-terminal, C-terminal, and loop regions are more prone to movements, compared with the residues forming the β-sheets (Fig. S3*F*). To test whether a specific geometry of arrangement between WW1 and WW2 is required to generate the binding pocket, we asked whether the isolated WW domains can directly bind to each other or to the WW1+WW2 domain. GST pulldown showed that neither WW1, WW2, nor the WW1 + 2 domains interact with either the isolated WW2 domain or the WW1 + 2 domain (Fig. S4*A*). Furthermore, the WW2 domain did not, when isolated, promote the interaction of the WW1 domain with PDZD11 (Fig. S4, *B* and *C*). Together, these data indicate that the tandem WW domains of PLEKHA7 do not dimerize, but interact weakly, when in a specific spatial geometry, through a small complementary surface, and Asp-30, Thr-35, and His-75 drive the creation of a pocket that allows high-affinity binding to PDZD11 (Fig. 2, *C* and *D*). Interestingly, the residue His-75 and even more so the Thr-35 are well conserved within tandem WW domains (Fig. S5, *A*–*C*), suggesting that they may play a similar role in stabilizing potential interactions between tandem domains of additional proteins.

**Figure 4.**
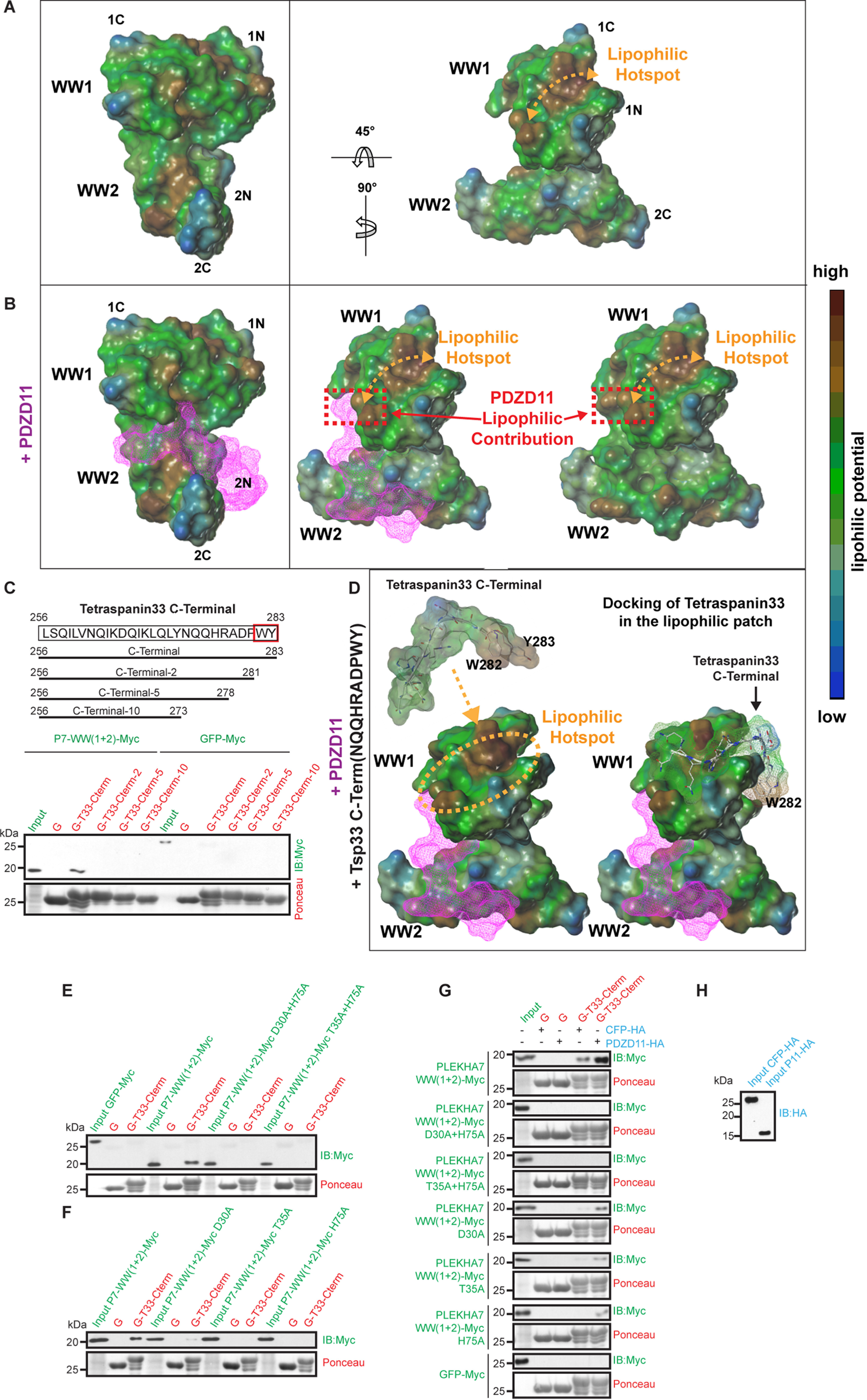
**Binding of PDZ11 to the WW1 + WW2 domains induces an increase in the size of a hydrophobic hot spot region on WW1, which docks the tetraspanin 33 C-terminal peptide.** *A* and *B,* lipophilic potential of the surface of the WW1–WW2 domains in the absence (*A*) or presence (*B*) of PDZD11. PDZD11 (16-27 peptide) is represented in *magenta*, with a wide frames surface. The image shows the molecule from different space orientations turning along the *y* (+90°) and *x* (−45°) axis. An *orange double arrow* indicates the hydrophobic hot spot region of the WW1 domain. In the presence of PDZD11 the surface of the lipophilic surface is increased, thus potentially enhancing the binding of hydrophobic ligands. The image was generated using the Molcad tool from SybylX-2.1.1. °C. *Top,* schemes of truncated constructs of Tspan33 used in GST pulldown assays. *Bottom,* immunoblot analysis of GST pulldowns using GST (*G*) fused to either C-terminal Tspan33 (*G-T33-Cterm*) or the truncated constructs (shown on *left*) as baits, and either PLEKHA7-WW(1 + 2)-Myc or GFP-Myc as preys. *D,* binding of the C-term dodecapeptide Tspan-33 (NQQHRADFWY) on the lipophilic surface of the WW1 domain. The WW1–WW2-PDZD11 complex is represented as surface with the WW1–WW2 domains as lipophilic potential and PDZD11 as *magenta* wide frames. The Tspan33 peptide is represented by *gray capped sticks* and a transparent lipophilic surface, where the Tyr and Trp residues constitute a highly hydrophobic part of the peptide. On the *right*, molecular docking shows how the Tspan33 peptide perfectly fits the lipophilic surface. *E* and *F,* single and double mutants of the WW1 domain fail to bind to Tspan33. Immunoblot analysis of GST pulldowns using either GST or GST (*G*) fused to Tspan33 C terminus (G-T33-Cterm) as baits, and either PLEKHA7-WW(1 + 2)-Myc WT and mutant or GFP-Myc as preys. *G* and *H,* PDZD11 promotes binding of the single but not double mutants of WW1 + WW2 to Tspan33. Immunoblot analysis of GST pulldowns using either GST or GST-Tspan33 baits (indicated in *red*), with either WT or single or double mutant WW1 + WW2 preys (*green*) Ponceau S-stained blots show the amounts of recombinant proteins used as bait. The third protein (either PDZD11-HA or CFP-HA, negative control) for tri-molecular pulldowns is shown in *blue* (normalization shown in *H*).

### *The* N-terminal *polyproline sequences of PDZD11 are required for interaction with PLEKHA7*

Next, we sought to identify the sequences within PDZD11, which are required for its interaction with PLEKHA7. Previously, we showed that the isolated N-terminal domain of PDZD11 (P7-ID: P7-interaction domain, residues 1-44) is sufficient for the interaction with the WW1 domain of PLEKHA7 (15). Thus, we generated a series of deletion mutants of the P7-ID, within full-length PDZD11 and analyzed their ability to interact with full-length PLEKHA7 by GST pulldown (Fig. 3*A*). Immunoblot analysis showed that deletion of up to 14 N-terminal residues (Δ4, Δ9, Δ14) of full-length PDZD11 did not impact the binding to PLEKHA7, when compared with WT PDZD11, whereas deletion of 19, 24, or 29 residues (Δ19, Δ24, and Δ29 constructs) resulted in either decreased or no interaction above background (Fig. 3*A*). Furthermore, a bait construct consisting of the first N-terminal 30 residues of PDZD11 was sufficient to interact with the PLEKHA7 prey (Fig. 3*A*, *N-term 1-30*). This analysis suggested that residues 16-30 of PDZD11 are essential for its interaction with PLEKHA7.

To identify more precisely the residues of PDZD11 involved in interaction with PLEKHA7 we performed mutagenesis of residues within the 16-30 PDZD11 sequence. Analysis of the sequence conservation of the N terminus of PDZD11 in 100 vertebrate species revealed that 27/31 residues are identical, including 5 proline residues, 4 of which (Pro-21, -22, -26, and -27) in the form of two consecutive prolines (Fig. 3*B*, *red box*). Because polyproline sequences are key ligands for WW domains (22), we generated either single or multiple alanine mutants of these prolines, at positions 16, 21, 22, 26, and 27 (Fig. 3*B*, *yellow highlight*). In addition, we also generated alanine mutations of the highly conserved Tyr-18, Glu-19, and Asn-20 residues (Fig. 3*B*, *yellow highlight*). GST fusion proteins were generated either in the context of a short construct of the first 30 residues of PDZD11 (Fig. 3*C*), or in the context of full-length PDZD11 (Fig. 3*D*), this latter to provide the binding region with the stabilizing effect of the downstream sequence. The ability of these fusion proteins to interact with the PLEKHA7 prey was examined by GST pulldown (Fig. 3, *C* and *D*). Immunoblot analysis showed that the E19A,N20A, and P26A + P27A mutants interacted with PLEKHA7 similarly to WT (Fig. 3, *C* and *D*). The P16A and P21A,P22A mutants showed reduced interaction with PLEKHA7 only when in the context of the short (1–30) construct (Fig. 3*C*), but not in the context of full-length PDZD11 (Fig. 3*D*). In contrast, the mutation Y18A and the combined mutations of either P16,21,22A, or P21,22,26,27A, or P16,26,27A or P16,21,22,26,27A resulted in either decreased or undetectable interaction (polyproline mutations) with PLEKHA7 both in the context of the short N-terminal construct, and in the context of full-length PDZD11 (Fig. 3, *C* and *D*). These results indicate that the residues that affect interaction with PLEKHA7 are either required to maintain the correct folding of the N-terminal domain of PDZD11 or are directly implicated in the interaction with PLEKHA7. Ponceau S staining of the blots showed that within the short N-terminal bait, the proline mutants but not the Y18A mutant migrated as two main polypeptides (Fig. 3*C*), whereas within the full-length molecule, they all migrated as a single band (Fig. 3*D*). This observation suggests that proline mutations destabilize the conformation of the short peptide, rendering it susceptible to proteolytic degradation, whereas when the mutations are in the context of the full-length molecule, the structure is stabilized.

Next, we asked whether the PDZD11 mutations influenced the junctional localization of exogenous PDZD11 in WT cells (Fig. 3*E*). In cells transiently transfected with GFP alone, GFP labeling was detected in cytoplasm (Fig. 3*E*, +*GFP*). In cells transfected with either GFP-tagged PDZD11 WT, or with the mutants P16A, E19A, N20A, P21,22A and P26,27A exogenous PDZD11, labeled by GFP, was colocalized with endogenous PLEKHA7 at junctions (Fig. 3*E*, *arrows*). The GFP-tagged construct of PDZD11 with the mutations at Y18A was localized both at junctions and in the cytoplasm (Fig. 3*E*, *arrows* and *asterisks*), whereas GFP-tagged constructs of PDZD11 with mutations at P16,21,22A, or P16,26,27A, or P21,22,26,27A, or P16,21,22,26,27A, were localized predominantly in the cytoplasm (Fig. 3*E*, *asterisks*). Together, these results identify the polyproline stretches 16, 21, 22, and 26, 27, together with Tyr-18, as the key residues of PDZD11 that are implicated in recruitment by PLEKHA7 at the junction in cells.

Finally, based on these experimental data, we docked the N-terminal heptapeptide of PDZD11 (residues 16 to 26) into the binding pocket of the WW1–WW2 model of PLEKHA7 (Fig. 3*F*). The experimental constraints used for docking are the identified interacting residues on WW1 and WW2. In the model, the peptide displays an N-terminal/C-terminal orientation similar to what is known for other proline-rich WW domain ligand (22) placing the prolines 21-22 in proximity of the pocket formed by Arg-33, Asp-30, and His-75. Furthermore, we observed that Tyr-18 can drive the interaction with the WW1 domain in two possible orientations. In the first, Tyr-18 forms an interaction with Arg-21 either through a cation-π interaction with the charged residue's side chain (Fig. 3*F*) or with a hydrogen bond (Fig. S5*D*, *violet model*). In the second mode, the peptide shifts toward the right, and the Tyr-18 can form a hydrogen bond with the backbone of Arg-33 (Fig. S5*D*, *pink model*). Our experimental data do not test the existence or relative populations of these modes. The PDZD11 peptide also interacts with WW2, reinforcing the overall complex stability. The tandem prolines 26-27 and Trp-24 are accommodated in the hydrophobic and solvent-exposed face (formed by Phe-64 and Phe-72) of the WW2 domain (Fig. 3*F*). Finally, the stability of the complex was monitored measuring the central mass distance between the PDZD11 peptide (residues 16-22) and, respectively, WW1 and WW2 domains during 120 ns of MD simulation (Fig. S5*E*). The PDZD11 peptide strongly maintains the interaction with the WW1 domain, but not the one with WW2, which is restored only in the final steps of the simulation (Fig. S5*E*). Although the short MD simulation does not permit full evaluation of large scale motions and populations of side chain rotamers, these results suggest that the binding of PDZD11 to the tandem WW domains can cause a conformational rearrangement of the tandem WW1–WW2 domains, which can favor the successive binding of other effector proteins, as Tspan33.

### PDZD11 promotes the interaction of Tspan33 with PLEKHA7 by increasing a hydrophobic surface on the WW1 domain

By GST pulldown analysis, the N-terminal region of PLEKHA7 binds weakly to the C-terminal region of Tspan33, but this interaction is greatly enhanced in the presence of PDZD11 (13). In agreement, PDZD11 is required in cells for the interaction of PLEKHA7 with Tspan33, and clustering of the Tspan33-ADAM10 complex at junctions (13). To investigate the mechanism through which PDZD11 promotes binding of Tspan33 to the WW domains of PLEKHA7 using the interaction model developed above, we stripped off the PDZD11 ligand to investigate the lipophilic molecular surface properties of the WW1–WW2 complex either in the absence (Fig. 4*A*) or presence (Fig. 4*B*) of PDZD11. PDZD11 (Fig. 4*B*, *purple*) binds to the pocket generated at the interface between WW1 and WW2 domains (see also Fig. 3*F*). We noticed that the WW1 domain displays a highly lipophilic region at the solvent-accessible surface, which resulted in a “hydrophobic hot spot” for protein recognition (Fig. 4*A*, *right, orange arrow*). The lipophilic cavity is formed mainly by the conserved residues Leu-10, Trp-15, Tyr-17, Phe-27, Leu-32, and Leu-38, which are not directly involved in the interaction with PDZD11. Strikingly, the presence of PDZD11 significantly increased (by 73.26 Å^2^) the size of this lipophilic surface and thus, extending the hydrophobic hot spot region (Fig. 4*B*, *right, red dotted rectangle*). Previously, we showed that the isolated C-terminal domain of Tspan33 (residues 256-283) is sufficient for the interaction with the WW1 domain of PLEKHA7 (13). Here we generated three deletion mutants (Δ2, Δ5, Δ10) of the C-terminal tail of Tspan33 and analyzed their ability to interact with the WW1 + WW2 domains of PLEKHA7 by GST pulldown (Fig. 4*C*). Immunoblot analysis showed that deletion of the last 2 residues of Tspan33 (Trp-Tyr) was sufficient to abolish the interaction between Tspan33 and PLEKHA7 (Fig. 4*C*). Interestingly, the C-terminal region of Tspan33 presents an excellent complementarity in terms of lipophilic surface with respect to the hydrophobic hot spot region of the WW1 domain (Fig. 4*D* left). The docking of the C-term Tspan33 decapeptide perfectly places the last two residues, Trp-282 and Tyr-283, in the hydrophobic region of the WW1 domain (Fig. 4*D*, *right*). Indeed, Trp-282 and Tyr-283 are the main contributors to the lipophilic surface of the Tpsan33 C-term, suggesting that the recognition of these macromolecules (WW1–WW2-PDZD11 and Tspan33) is mediated especially by hydrophobic/hydrophilic forces. To confirm the role of these residues, we studied mutants of PLEKHA7, and examined how double and single mutations within the WW1 and WW2 domains affect interaction with the C terminus of Tspan33 by GST pulldown. Although the GST fusion of the last 28 residues of Tspan33 (G-T33-Cterm, Fig. 4*E*) interacted well with the WW1 + WW2 prey, either the double mutations D30A+H75A or T35A+H75A (Fig. 4*E*) or the single mutations D30A, T35A, and H75A (Fig. 4*F*) abolished binding. This suggested that the lipophilic surface of WW1 that interacts with Tspan33 is exquisitely sensitive to the conformation of the tandem domain structure, and may require PDZD11 for stabilization. To test this, we compared the binding of the WW1 + WW2 prey to the Tspan33 C-terminal bait in the absence or presence of either PDZD11 or CFP, as a negative control (Fig. 4, *G* and *H*). Immunoblot analysis showed that PDZD11 increases the binding of both WT and single mutant preys to the Tspan33 bait, but cannot rescue the loss of binding to the double mutant prey (Fig. 4, *G* and *H*), consistent with the observation that it does not bind to the double mutant (Fig. 2*A*).

## Discussion

The precise molecular mechanisms through which PLEKHA7 is implicated in disease (5, 6), morphogenesis (8, 23), and diverse cellular functions (9–11) are poorly understood. Here we address the structure-function relationships of the N-terminal tandem WW domains of PLEKHA7, a critically important region for the ability of PLEKHA7 to scaffold and cluster transmembrane proteins (13, 15). We propose a structural model (Movie S1), which provides a rational explanation for the ability of the WW2 domain of PLEKHA7 to promote binding of the WW1 domain to PDZD11, and for the ability of PDZD11 to promote Tspan33 binding to the WW1 domain. Our model assumes a single rigid conformational state, which as noted above requires additional experimental testing.

Proteins that contain multiple or tandem WW domains show different structural configurations and reciprocal functional interactions between these domains (24, 25). In some cases tandem WW domains are separated by a relatively rigid helical linker, and their aromatic polyproline-binding surfaces show opposite orientations, allowing interaction with distinct ligands (26). In other cases there is a greater degree of freedom between the two WW domains, allowing different spatial orientation of ligands and substrates (27). An additive effect can occur when both WW domains can interact with the same ligand, and the binding affinity to one is increased by the presence of the second domain (24, 25). The second of the tandem WW domains can act as a chaperone, to facilitate ligand binding to the first domain, whereas not interacting with the ligand, or can change either the conformation or ligand binding specificity of the second WW domain. Tandem WW domains can also form a cleft at their junction, which allows ligand binding to both domains (28–34). Our results indicate proteolytic degradation of the isolated bacterially expressed WW1 domain constructs, whereas the same constructs are not degraded when in the context of the WW1 + WW2 sequence, suggesting that the presence of the WW2 domain stabilizes the structure of the WW1 domain. The WW2 domain may also act by modifying the conformation of the WW1 domain ligand-binding groove, because none of the WW1 mutations that prevented the interaction of the isolated WW1 domain with PDZD11 was effective when in the context of the tandem WW1 + WW2 domain. Another, nonexclusive possibility, is that binding of the ligand to the WW2 domain occurs only when in the context of the WW1 + WW2 domains, and contributes to increasing the binding affinity to the WW1 domain for either the same ligand, or for a different ligand. Conversely, binding of PDZD11 to the WW1 domain may synergistically enhance the PDZD11 binding potential of the WW2 domain, as has been suggested for other tandem WW proteins (24). The relatively high sequence identity between the WW1 and WW2 domains of PLEKHA7 suggests that they could both bind to the same ligand. By exploring how combined mutations in both WW1 and WW2 domains affect the interaction with PDZD11, and modeling the structure of the PLEKHA7 WW domains, we propose that Asp-30 of the WW1 domain interacts with His-75 of WW2. In our model, which needs to be further validated by either NMR spectroscopy or other computational and biophysical methods, the contact surface between the two WW domains is complementary, but relatively small (549 Å^2^), and these domains form a binding pocket that interacts with the N-terminal, proline-rich peptide of PDZD11. Three residues in the WW1 domain, Arg-21, Asp-30, and Thr-35, and one residue in the WW2 domain, His-75, are critically involved in promoting the interaction of PLEKHA7 with PDZD11.

WW domains are classified based on the sequences of the interacting ligands in 4 groups: PPXY (I), PPLP (II), PR (III), or p(S/T)P (IV). The sequence of the WW-interacting region of PDZD11 does not precisely match any of these sequences. However, group II and group III WW domains not only recognize PPLP and PR-containing peptides, but also polyproline stretches containing glycine, methionine, or arginine (20). In addition to the polyproline sequence of the N-terminal region of PDZD11, the Tyr-18 residue of PDZD11 also appears crucial in the binding to the WW domains, probably guiding the orientation of the peptide and interacting with either Arg-21 or Arg-33. Moreover, the tandem prolines Pro-26–Pro-27 of PDZD11 appear difficult to pinpoint in the interaction with the tandem domains, as shown by GST pulldowns. Nevertheless, their accommodation in the solvent-exposed face of WW2 domain might contribute to strengthening the overall stability of the complex.

The PDZD11-dependent clustering of transmembrane proteins by PLEKHA7 appears to occur by at least two different mechanisms. In the case of nectins, their C terminus interacts with the PDZ domain of PDZD11 through a canonical PDZ-binding motif (15), indicating that PLEKHA7 anchors nectins indirectly, through PDZD11. However, in the case of Tspan33, the C-terminal region of Tspan33 interacts directly with the WW1 domain of PLEKHA7, but not with PDZD11 (13). The C-terminal sequence of Tspan33 contains a proline residue (Asp-Pro-Trp-Tyr) but does not fall into any of the previously established ligand groups for recognition by WW domains, making it unlikely that it is a canonical binding mode. In agreement, based on the lipophilic surface properties, the C-terminal Tspan33 sequence can recognize a highly hydrophobic and solvent-exposed region of WW1 that is distinct from the one that interacts with PDZD11. The binding of PDZD11 to the cleft between the WW1 and WW2 domains not only stabilizes the WW1 + WW2 tandem domains, but also contributes to increasing the surface of the lipophilic region, which is a perfect “hot spot” for proteins recognition driven by hydrophobic forces (35). Molecular docking shows that the Trp-Tyr dipeptide at the extreme C terminus of Tspan33 perfectly fits into the extended lipophilic surface.

Our molecular studies provide a rationale explaining how PDZD11 promotes the binding of the C terminus of Tspan33 to PLEKHA7. Previously we showed that clustering of ADAM10 at junctions occurs through sequential and hierarchical multimolecular interactions. ADAM10 must first be docked to junctions through the interaction of its chaperone Tspan33 with the PLEKHA7-PDZD11 complex, before it can be “locked” by direct interaction with afadin (13). Here we show a new mechanism, whereby the conformational change driven by PDZD11 binding to a specific binding pocket allosterically modifies the conformation of the WW1 domain, to promote interaction with hydrophobic amino acids of a transmembrane ligand. It is interesting that PDZD11 binds to the C terminus of the sodium-dependent multivitamin transporter, and this latter is stabilized by Tetraspanin-1 (Tspan-1) (36). Future studies should investigate whether PLEKHA7 is involved in anchoring of the multivitamin transporter or other PDZD11-binding transmembrane proteins (37, 38) to junctions, through mechanisms similar to the one described here.

In summary, we characterize the structural basis of the interaction between the tandem PLEKHA7 domains and their interactions with both PDZD11 and Tspan33. Our model illustrates the mechanism through which the PLEKHA7-PDZD11 complex forms a scaffold for a transmembrane ligand with hydrophobic C-terminal sequences. In this model, cooperative interaction between WW1 and WW2 promotes binding of WW1 to PDZD11, and this in turn promotes Tspan33 binding to a different surface of WW1. This mechanism may be relevant to the functions of additional proteins with tandem WW domains.

## Experimental procedures

### Cell culture and transfection

Mouse cortical collecting duct cells (mCCD clone N64-Tet-off, a kind gift from Prof. Eric Feraille, University of Geneva, Switzerland) were grown in Dulbecco's modified Eagle's medium (Gibco) supplemented with 20% heat-inactivated fetal bovine serum (FBS; PAN Biotech), 1× minimal essential medium nonessential amino acids (NEAA; Gibco). PLEKHA7-KO mCCD cells were obtained by CRISPR/Cas9 genomic editing (13). HEK cells were cultured in the same medium, supplemented with 10% FBS. Transfections were performed using Lipofectamine 2000, following the manufacturer's guidelines (Thermo Fisher Scientific).

### Antibodies

Primary antibodies were: polyclonal guinea pig anti- PLEKHA7 (in-house gp2737, 1:500 IF); monoclonal mouse anti-GFP (Roche Applied Science, 11814460001, 1:100 IF), polyclonal rabbit anti-PDZD11 (in-house r29958 28, 1:50 IF), mouse anti-HA (Zymed Laboratories Inc., 32-6700, 1:1000 IB), and mouse anti-His (Invitrogen, catalog number 37-2900, 1:1500 IB). Secondary antibodies were anti-mouse Alexa Fluor 488, anti-rabbit Alexa Fluor 488, anti-rabbit Cy3, anti-mouse Cy3, and anti-guinea pig Cy3 (Jackson ImmunoResearch Europe, 1:250 IF), and HRP-conjugated anti-mouse (Promega, 1:20000, IB).

### Plasmids and mutagenesis

Constructs of pTre2-Hyg containing GFP-Myc, GFP- PLEKHA7 (human) full-length (residues 1-1121), PLEKHA7-WW(1 + 2)-Myc (residues 1-162), PLEKHA7-WW(2)-Myc (residues 50-162), GFP-PDZD11 (human) full-length (residues 1-140), and constructs of pcDNA3.1 containing CFP-HA, PDZD11-HA (human) full-length, and constructs of pFASTBAC containing PLEKHA7-His and constructs of pGEX-4T1 containing GST alone, GST-PLEKHA7 (human) WW1 domain (residues 1-56), WW1 + WW2 domain (1-162), WW2 domain (50-162), GST-PDZD11 full-length (residues 1-140), and GST-Tetraspanin 33 C-terminal (residues 256-283) were described previously (13, 15). Mutations into alanine were performed using Q5 Site-directed Mutagenesis Kit, following the manufacturer's guidelines (New England BioLabs). Mutant constructs of PLEKHA7 and PDZD11 were generated by PCR amplification on inserts of either GFP-PLEKHA7 full-length in pTre2Hyg vector or GFP-PDZD11 full-length in pcDNA3.1 vector (15), using the appropriate oligonucleotides. The sequence of all mutated constructs was verified, to rule out off-target mutations. For bacterial expression, mutated sequences were subsequently amplified and cloned either into the BamHI-NotI sites (PLEKHA7) or EcoRI-NotI sites (PDZD11) of pGEX-4T1 vector. Deletion constructs of PDZD11 and Tetraspanin 33 C-terminal were generated by PCR amplification with the appropriate oligonucleotides cloned either into the EcoRI-NotI sites (PDZD11) or BamHI-NotI sites (Tetraspanin 33 C-terminal) of pGEX-4T1 vector.

### Recombinant protein expression and GST pulldowns

For the production and purification of GST fusion protein baits, *Escherichia coli* (BL21-DE3) were induced with 0.1 mm isopropyl 1-thio-β-d-galactopyranoside (2 h at 37 °C). Bacterial pellets after centrifugation were lysed in PBS containing 1% Triton X-100, 5 mg/ml of antipain, 5 mg/ml of leupeptin, 5 mg/ml of pepstatin, 1 mm phenylmethylsulfonyl fluoride, and cell debris were removed by centrifugation at 13,000 rpm (15 min at 4 °C). The supernatants containing GST-tagged proteins were normalized for protein content by SDS-PAGE. For expression of prey proteins in mammalian cells, 2 × 10^6^ HEK cells were plated in a 100-mm^2^ dish and transfected with 20 μg of the HA-tagged construct, lysed 2 days after transfection using co-IP buffer (150 mm NaCl, 20 mm Tris-HCl, pH 7.5, 1% Nonidet P-40, 1 mm EDTA). Expression of PLEKHA7 in Sf9 insect cells (Sf9) was as described in Ref. 15. Prey proteins were normalized by immunoblotting with antibodies against tags (HA, His, and Myc). For GST pulldowns 5 μg of bait GST fusion protein was coupled for 1 h at room temperature to 10 μl of GSH-Sepharose beads. Following incubation and 3× washing with PBS containing 2% BSA and 1% Nonidet P-40, the beads were incubated for 1 h at 4 °C with normalized lysates of either HEK or insect cells. Proteins bound to the beads were eluted with 20 μl of SDS sample buffer at 95 °C for 5 min, and 10 μl of eluate was loaded on SDS gels.

### Immunofluorescence

Cells were seeded at density of 40,000 cells/well onto 12-mm glass coverslips in 24-well-plates (Falcon^TM^ Polystyrene Microplates). 24 h after seeding cells were transfected, and 48 h later they were fixed with methanol (6 min at −20 °C), rehydrated in PBS (3×), permeabilized with 0.3% Triton X-100 in PBS for 5 min, and incubated with 0.03% Triton X-100, 1% BSA, 0.2% gelatin in PBS for 20 min. Cells were then incubated with primary antibodies (2 h at room temperature), washed with PBS, and incubated with 0.03% Triton X-100, 1% BSA, 0.2% gelatin in PBS for 15 min and incubated with secondary antibodies (1 h, at room temperature), washed 2× with 0.03% Triton X-100, 1× with cold PBS, and 1× with water. Coverslips were mounted with Vectashield containing DAPI (Reactolab).

### SDS-PAGE and immunoblotting

For preparation of cell lysates, cells were washed with cold PBS, lysed with co-IP buffer with Roche inhibitor (1×) at 4 °C, and incubated for 15 min with gentle agitation. Lysates were sonicated 5 s at 66% power (3 bursts), centrifuged 20 min at 13,000 rpm, and supernatants were recovered. Proteins were mixed with SDS sample buffer and incubated at 95 °C for 5 min. Inputs of prey protein lysates and baits in bacterial lysates were normalized by analysis on SDS gels (8–12% acrylamide, 100 V). For immunoblots, gels were transferred onto nitrocellulose (0.45 μm) (100 V for 80 min at 4 °C), and blots were incubated with primary antibody (anti-HA, anti-Myc, or anti-His), followed by secondary HRP-labeled antibody (1:20,000), and visualized with ECL (Amersham Biosciences).

### Sequence analysis

To analyze protein sequence conservation, the sequences of the WW domain of PLEKHA7 or PDZD11 from all available vertebrate species (*n* = 100) were retrieved with blastp, using either the human PLEKHA7 or human PDZD11 sequences as a query. Sequences were aligned using Weblogo (RRID:SCR_010236).

### Homology modeling

The Prime program from Schrödinger package (Prime, Schrödinger, LLC, New York, NY, 2016) was used to generate the homology models of WW1 and WW2 domains. The selection of the corresponding template for building the models was based on the sequence similarity study performed with BLAST (basic local alignment search tool). The solution structure of Nedd4 WW domain (39) was chosen for building the WW1 domain, whereas the WW domain of the human syntaxin-binding protein 4 (40) was used for the WW2 model. Ramachandran plots were then used to assess the correct geometry of the amino acids residues as confirmation of the quality of the model (RRID:SCR_017590).

### Molecular docking

The homology models were submitted to the Protein Preparation Wizard of Schrödinger tools package, where bond orders and atom types were assigned and also terminals were capped. The p*K_a_* values were predicted using PROPKA at neutral pH and finally an all-atoms restrain minimization with a maximum RMSD of 0.3 Å was performed. The amino acids and the peptide sequences used for docking were prepared with the LigPrep tool that generates different energetically favored conformations, considering also the possible protonation states of the ligands. Glide program from Schrödinger package (Schrödinger Release 2016-4: Glide, Schrödinger, LLC, New York, NY) was used to perform the molecular docking. The calculation considers a predefined target grid-box, and in the case of PDZD11 peptide docking we centered the box at the level of residues Asp-30, Arg-33, and Thr-35 for the WW1 domain, and His-75 for the WW2 domain. In the case of Tspan33 docking, the grid was centered in the lipophilic region of the WW1 domain considering the residues (Leu-11, Trp-15, Tyr-17, Phe-27, Leu-32, and Ile-38). Finally, the standard-precision mode was considered in the case of docking single amino acid residues and the SP peptide-mode was chosen for the peptides (Schrödinger Release 2016-4: Glide, Schrödinger, LLC, New York, NY).

### Molecular dynamic simulations

The Amber16 suite programs (21) was used to perform molecular dynamic simulations, respectively, 160 ns for the WW1–WW2 complex and 120 ns for the WW1–WW2-PDZD11 complex, WW1–WW2 single mutants (T35A, D30A) and WW1–WW2 double mutant (T35A,H75A and D30A,H75A) complex. The WW1–WW2 complex representing the putative interacting mode of the two domains was chosen as a starting structure for the dynamic calculation. The Amber ff14SB force field was used to parametrize the two structures using the Leap program from Amber's package. The systems were then solvated with TIP3P water into a cubic box of 12 Å and neutralized with sodium as counter ion. The Build panel of the Maestro interface (Schrödinger Release 2016-4: Maestro, Schrödinger, LLC, New York, NY) was used to mutate the corresponding residue. All complexes then underwent minimization, heating, and equilibration protocols: (i) a first 5000-step minimization step, where restrains were applied to the amino acid residues (steepest descendent and conjugated gradient methods were applied), followed by a second 5000-step minimization where the entire system was allowed to move, using the Sander program. (ii) During the heating phase the temperature increases from 0 to 298 K using the Langevin thermostat, and with 100 kcal mol^-1^ Å^-2^ positional restrains for protein and ions over the course of the first 80 ps at constant pressure (1 atm). The restrained forces were gradually turned off to zero in the subsequent 100 ps, allowing movement of all atoms. (iii) A final heating and equilibrating step was performed at 300 K at constant pressure over 820 ps. Finally, the molecular dynamic production was performed under NTP ensemble (constant pressure, 1 atm; and temperature, 300 K) using the PMEMD Cuda. The SHAKE algorithm was used to restrain the lengths of all bonds involving hydrogen atoms, allowing an increase of the time step up to 2 fs with a cut-off space of 9 Å. The default particle-mesh Ewald settings (which correspond to a grid spacing of ∼1 Å and a direct space tolerance of 10^−6^) were used to determine long-range charge interactions. The CPPTRAJ of Amber16 was used to process and analyze the molecular dynamic results. The surface area at the interface of the WW1 and WW2 domains was determined on the basis of the difference between the solvent accessible surface areas of the two domains separately (WW1 + WW2) or in a complex (WW1–WW2).

## Data availability

Data and materials are available upon request from S.C. (sandra.citi@unige.ch), except for molecular docking and molecular dynamic simulation datasets, SRR9822082 which are available upon request from L.S. (leonardo.scapozza@unige.ch).

## Supplementary Material

Supporting Information
