# Supplementary material for: Cooperative binding of the tandem WW domains of PLEKHA7 to PDZD11 promotes conformation-dependent interaction with tetraspanin 33
Source: J Biol Chem. 2020 May 5;295(28):9299–312. doi: 10.1074/jbc.RA120.012987 (PMC7363125; doi:10.1074/jbc.RA120.012987)
Supplement: Supporting Information [file supp_295_28_9299__index.html]

Cooperative binding of the tandem WW domains of PLEKHA7 to PDZD11 promotes conformation-dependent interaction with tetraspanin 33 — Cooperative PLEKHA7-PDZD11-Tspan33 interaction — Cooperative binding of the tandem WW domains of PLEKHA7 to PDZD11 promotes conformation-dependent interaction with tetraspanin 33 — Cooperative PLEKHA7-PDZD11-Tspan33 interaction — Supporting Information 

# Cooperative binding of the tandem WW domains of PLEKHA7 to PDZD11 promotes conformation-dependent interaction with tetraspanin 33

## Supporting Information

- Supporting Information Movie 1 - Supporting Information Movie 1
- Supporting Information (to be published online) - SupportingInformationFiguresandLegends
