# Supplementary material for: Cooperative binding of the tandem WW domains of PLEKHA7 to PDZD11 promotes conformation-dependent interaction with tetraspanin 33
Source: J Biol Chem. 2020 May 5;295(28):9299–312. doi: 10.1074/jbc.RA120.012987 (PMC7363125; doi:10.1074/jbc.RA120.012987)
Supplement: Supporting Information [file supp_RA120.012987_158754_2_supp_524735_q9v2g3.pdf]

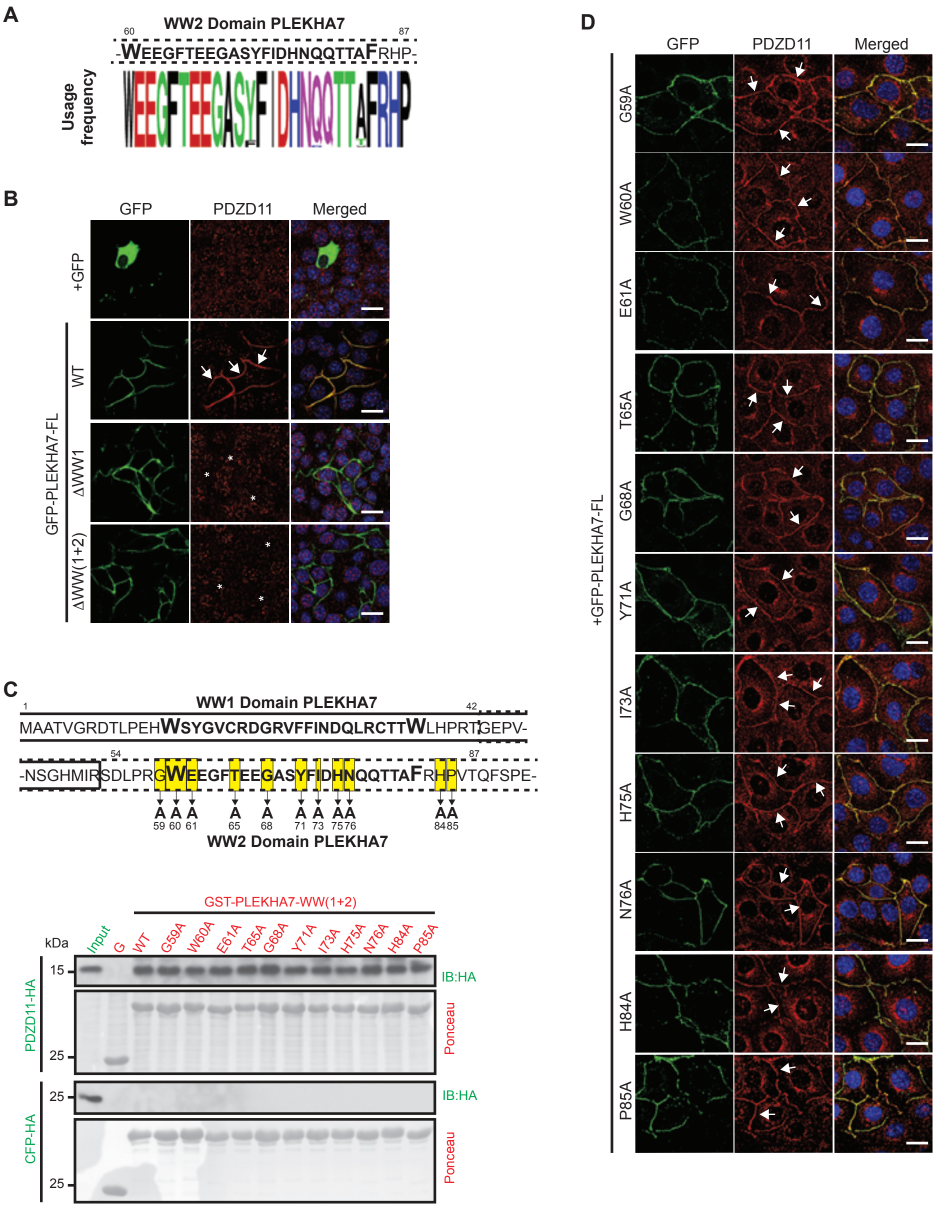


Supporting Information Figure 1

**SUPPORTING INFORMATION FIGURE 1.** Mutations within the WW2 domain of PLEKHA7 do not affect its interaction with PDZD11. A. Top: sequence of the WW2 domain of PLEKHA7, and bottom: Weblogo diagram of residue conservation. B. Requirement for the WW1 domain for PLEKHA7 interaction with PDZD11 in cells. Immunofluorescence localization of endogenous PDZD11 in PLEKHA7-KO mCCD cells rescued either with GFP, or WT GFP-PLEKHA7, or GFP-PLEKHA7 with N-terminal deletion of either WW1 (ΔWW1) or WW1+WW2 (ΔWW(1+2) domains). C. Top: sequence of WW1+WW2 domains, with mutations of highly conserved residues of WW2 (to Ala) highlighted in yellow. The numbers below each residue indicate residue number in the sequence. Bottom: immunoblot analysis of GST pulldowns using either GST (G), or GST fused to either WT or mutant WW1+WW2 domains as bait, and either PDZD11-HA or CFP-HA as preys. D. Immunofluorescence localization of endogenous PDZD11 in PLEKHA7-KO mCCD cells rescued with WW2 point mutants of GFP-tagged full-length PLEKHA7. Merged images show nuclei in blue (DAPI) and arrows indicate junctional labeling and asterisk reduced/undetectable labeling. Bar= 20 μm.

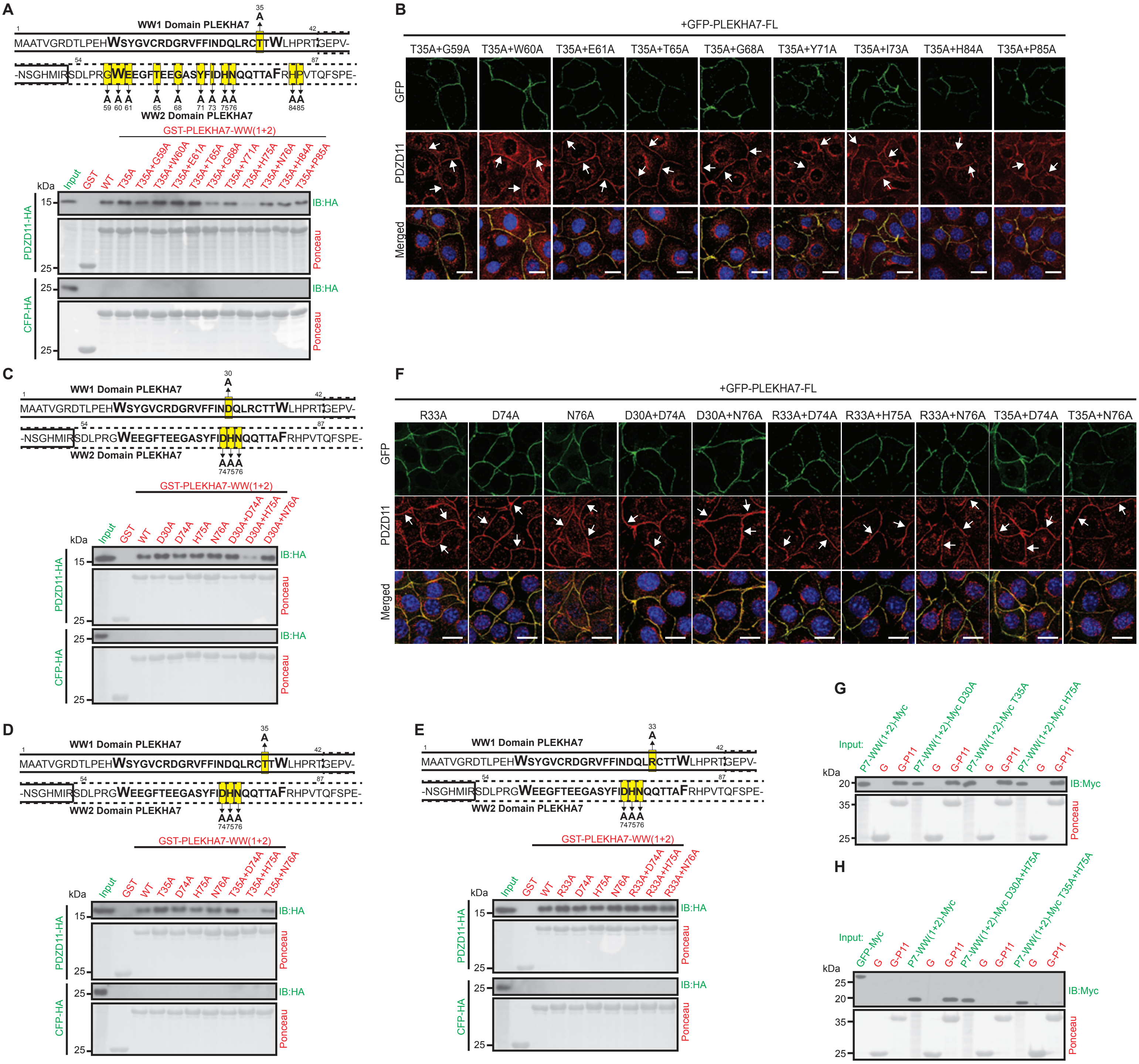


Supporting Information Figure 2

**SUPPORTING INFORMATION FIGURE 2.** (A, C, D, E) Top: sequence of WW(1+2) domains, with summary of each individual mutation within the WW2 domain used in different pulldowns, highlighted in yellow. The numbers below each residue indicate number in the sequence. Bottom: immunoblot analysis of GST pulldowns using either GST (G), or GST fused to WT and to each combination of WW1 and WW2 mutation as baits, and either CFP-HA or PDZD11-HA as preys. B, F. Immunofluorescence localization of endogenous PDZD11 in PLEKHA7-KO mCCD cells rescued either with GFP, or with either WT or the indicated combinations of point mutants within the WW1 and WW2 domains of GFP-tagged full-length PLEKHA7. Merged images show nuclei in blue (DAPI). Arrows indicate junctional labeling. Bar= 20 µm. G-H Single but not double mutants preys of WW1 and WW2 bind to the PDZD11 bait. Immunoblot analysis of GST pulldowns using either GST (G), or GST fused to full-length PDZD11 as baits, and either WT or indicated mutants of PLEKHA7-WW(1+2)-Myc or GFP-Myc (negative controls) as preys. (G) shows single mutant preys and (H) shows double mutant preys.

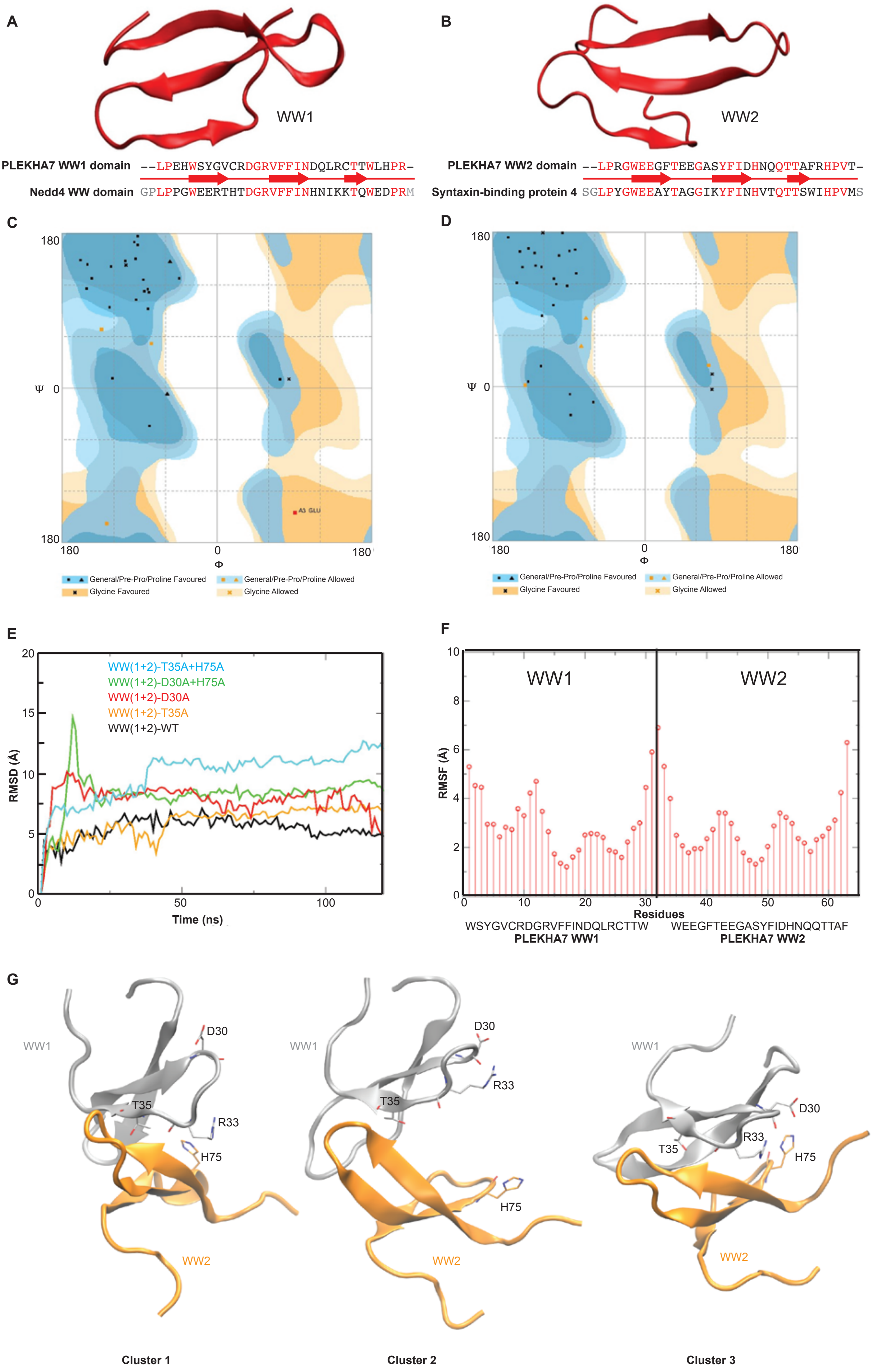


Supporting Information Figure 3

**SUPPORTING INFORMATION FIGURE 3.** Mutations D30A+H75A and T35A+H75A destabilize the triple β-sheet structure of the WW (1+2) domains. A. Top: Homology model of the WW1 domain of PLEKHA7, based ubiquitin ligase nedd4 (PDB code: 1I5H_W) as a template. Bottom: amino acid sequence alignment between the PLEKHA7-WW1 and the template. Identical residues are in red, and position of β-sheet secondary structure is indicated by arrows. B. Top: Homology model of WW2 domain based on syntaxin-binding protein (PDB code: 2YSG) as a template. Bottom: amino acid sequence alignment between the model and the template. C-D. Ramachandran plots of WW1 (C) and WW2 (D) domain models. For WW1, all amino acids are in the favored and allowed regions, respectively 25 and 3 residues, and only Glu at position 3 at the N-terminal is found in the outlier region. For WW2, 25 amino acids are in the favored regions and 4 in the allowed regions. E. Comparison of the RMSD values (120 ns trajectory) between the WT WW1-WW2 complex (black tracing) and the mutants D30A (red tracing), T35A (orange tracing), D30A+H75A (green tracing) and T35A-H75A (blue tracing). The x-axis represent the time-scale (120 ns) whereas in the y-axis there are the deviation values express in Å. F. Root mean square fluctuations (RMSF) of the WW1-WW2 complex, with the x-axis showing residue numbers of the two domains, and the Y-axis fluctuation values expressed in Å. G. Representative structures extracted from the cluster analysis of the 120 ns trajectory of WW1-WW2 complex. Cluster3 is the most populated cluster with an occurrence during the simulating time of 50%, whereas cluster 1 and 2 are presented respectively 18% and 19%. The remaining 13% of the clustered trajectory is composed by smaller clusters appearing with % of occurrence less than 4%.

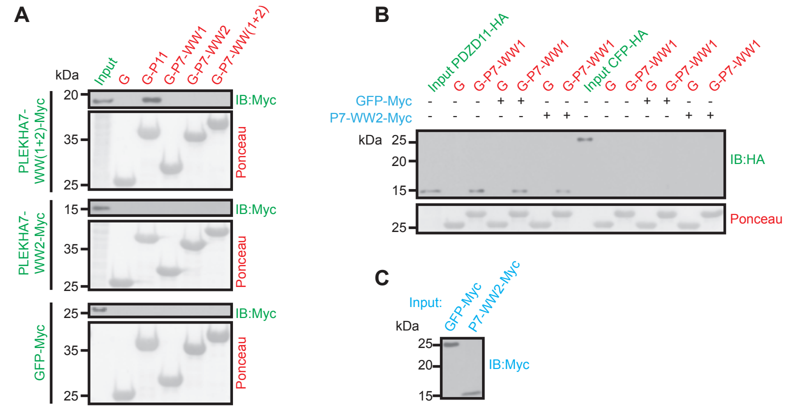


Supporting Information Figure 4

**SUPPORTING INFORMATION FIGURE 4.** Lack of interaction between WW1 and WW2 domains of PLEKHA7. (A). Immunoblot analyses of GST pulldowns using either GST (G) or GST fused to either WW1 (G-P7-WW1) or WW2 (G-P7-WW2), or both (G-P7-W(1+2) as baits, and either both WW1 and WW2 domains or only WW2 as preys. Preys were tagged with myc at the C-terminus. (B-C) Immunoblot analyses of GST pulldowns using either GST (G) or GST fused to WW1 (G-P7-WW1) as baits, and either PDZD11-HA or CFP-HA as a prey, either in the presence or in the absence of a third protein (either GFP-myc or the WW2 domain) as a third protein (trimolecular pulldown, third protein shown in blue). All preys were tagged with myc at the C-terminus. (C) shows normalization of third protein.

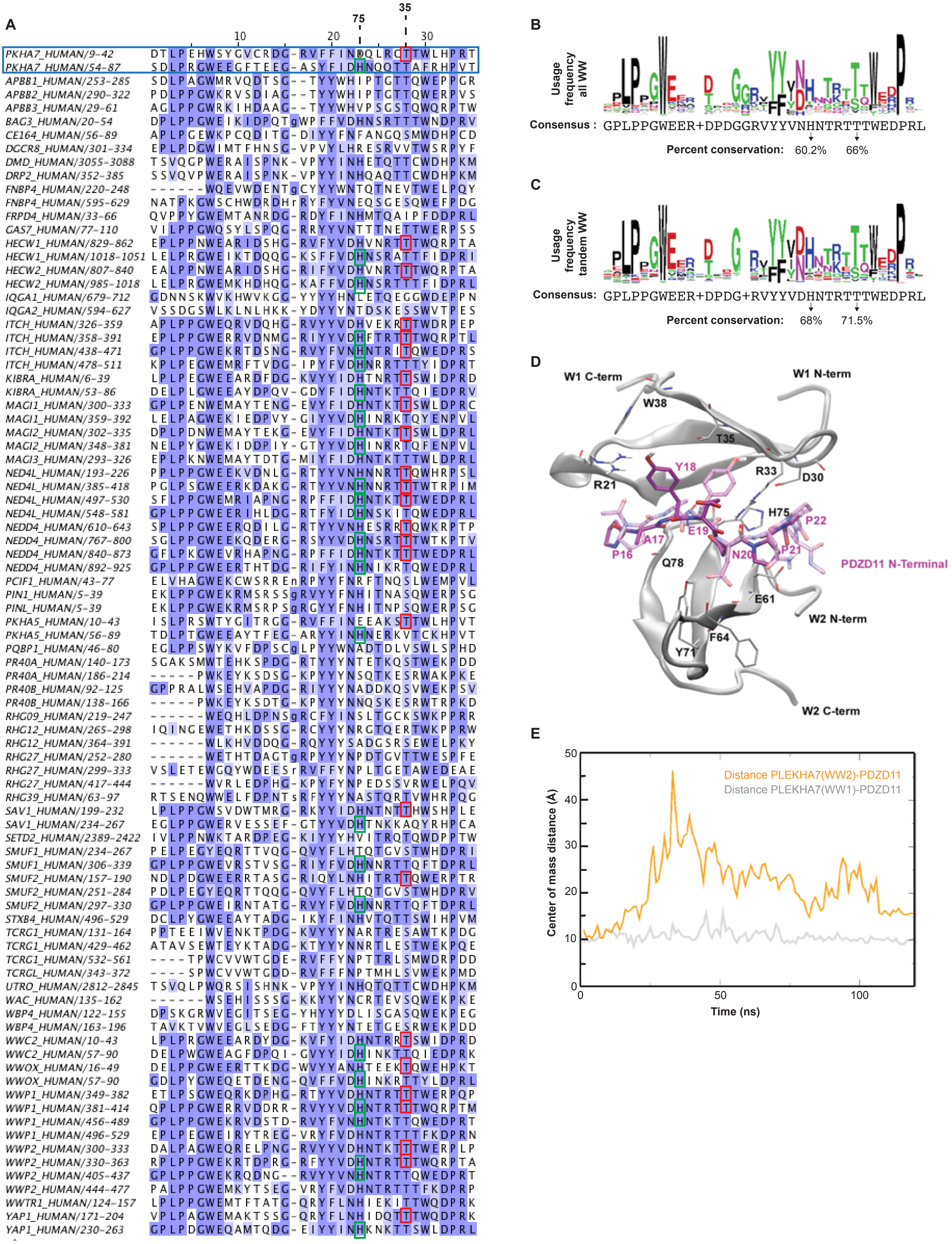


Supporting Information Figure 5

**SUPPORTING INFORMATION FIGURE 5**. Evolutionary conservation of T35 and H75 PLEKHA7 residues in single and tandem WW domains. A. The WW domains of human PLEKHA7 (in a blue box) (9-42, WW1 and 54-87, WW2) were aligned with 86 WW domains of human proteins. Light/dark grey highlighting indicates identical/similar residues. Conserved threonine residues at position 28 of the first WW in tandem WW domains are in a red box. Conserved histidine residues at position 22 of the second (or third) tandem WW domain are in a green box. B-C. Weblogo consensus sequences and usage frequency of single (B) and tandem (C) WW domains. The percent conservation of Thr at position 28 and His at position 22 is indicated. D. Two hypothetical (either pink or purple in the superposition model) modes of interaction between WW1-WW2 and PDZD11. The WW1 and WW2 domains are shown in grey as new cartoon style and the PDZD11 heptapeptide (PAYENPP) in magenta as licorice style. The image was generated with VMD software. E. The central mass distance calculated between PDZD11 and respectively versus WW1 (grey line) and WW2 (orange line) domains are shown as a measure of binding stability. The x-axis shows the simulated time-scale (120 ns) and the y-axis the distances values, expressed in Å.

| W1W2 complex | Ave RMSD (Å) |
|---|---|
| **WT** | 6.2 ± 0.81 |
| **D30A** | 6.5 ± 0.90 |
| **T35A** | 8.3 ± 1.02 |
| **D30A + H75A** | 9.2 ± 1.50 |
| **T35A + H75A** | 11.0 ± 1.92 |

**SUPPORTING INFORMATION TABLE 1.** The average values of the root-mean square deviations are summarized in the table for each simulated system.

| DONOR | ACCEPTOR | OCCUPANCY (%) |
|---|---|---|
| ARG33-Side | GLU66-Side | 7.74% |
| VAL19-Main | GLN77-Main | 4.16% |
| ARG33-Side | PHE64-Main | 0.42% |
| GLN78-Side | TYR17-Main | 4.91% |
| SER16-Side | GLN78-Side | 0.08% |
| ARG33-Side | TYR71-Side | 0.17% |
| ARG21-Side | THR80-Main | 0.67% |
| THR79-Side | VAL19-Main | 0.08% |
| GLN77-Side | TYR17-Side | 0.08% |
| CYS20-Side | THR79-Side | 0.17% |
| GLN77-Side | VAL19-Main | 0.25% |
| TYR71-Side | ARG33-Main | 0.08% |
| THR35-Side | TYR71-Side | 0.17% |
| CYS20-Side | THR80-Side | 0.50% |
| PHE64-Main | ARG33-Main | 23.48% |
| THR36-Side | GLU66-Side | 38.38% |
| THR35-Main | PHE64-Main | 7.74% |
| THR35-Side | PHE64-Main | 32.06% |
| ARG33-Side | GLU62-Main | 13.74% |
| ARG24-Side | GLU67-Side | 19.40% |
| LEU38-Main | GLU66-Side | 0.67% |
| TRP37-Side | GLU67-Side | 0.92% |
| TRP37-Main | GLU66-Side | 1.92% |
| ARG21-Main | THR80-Side | 0.08% |
| ARG33-Side | GLU61-Side | 19.73% |
| TYR71-Side | THR35-Side | 24.98% |
| ARG33-Side | GLU62-Side | 3.58% |
| CYS20-Side | TYR71-Side | 0.75% |
| TYR71-Side | CYS20-Side | 0.25% |
| THR80-Side | VAL19-Main | 2.50% |
| THR80-Main | VAL19-Main | 0.33% |
| ARG21-Main | THR80-Main | 1.08% |
| ARG11-Side | GLN53-Side | 0.58% |
| GLN78-Side | VAL19-Main | 1.50% |
| ARG21-Side | GLN77-Main | 1.17% |
| ARG21-Side | THR79-Side | 3.66% |
| CYS20-Side | GLN78-Main | 0.08% |
| ARG24-Side | THR80-Side | 2.00% |

| | | |
|---|---|---|
| ARG21-Main | GLN78-Main | 4.08% |
| TRP37-Side | GLU66-Side | 0.17% |
| CYS34-Side | THR65-Main | 0.08% |
| CYS20-Side | GLN78-Side | 0.08% |
| GLU66-Main | THR35-Main | 12.82% |
| ARG21-Side | ASN76-Side | 0.08% |
| ARG21-Main | GLN78-Side | 0.08% |
| ARG21-Side | ASN76-Main | 0.08% |
| ARG21-Side | HIP75-Main | 0.17% |
| GLN78-Side | ASP22-Side | 0.33% |
| ARG21-Side | GLN78-Side | 0.08% |
| ARG21-Side | GLN78-Main | 0.25% |
| ARG21-Side | TYR71-Side | 0.08% |
| ASP22-Main | GLN78-Side | 0.08% |
| THR80-Side | ASP22-Side | 1.08% |
| ARG21-Side | THR65-Side | 1.33% |
| THR65-Side | THR35-Side | 0.08% |
| CYS34-Side | GLU66-Side | 0.58% |
| ARG21-Side | GLU67-Side | 30.97% |
| VAL19-Main | TYR71-Side | 9.66% |
| SER16-Side | GLU61-Side | 10.32% |
| GLN78-Side | GLY18-Main | 0.42% |
| CYS20-Main | GLN78-Side | 0.08% |
| ARG21-Side | GLU66-Side | 0.08% |
| TYR17-Side | GLN78-Side | 0.08% |

**SUPPORTING INFORMATION TABLE 2**. The hydrogen bond frequency occurrence (%) calculated from the WW1-WW2 trajectory. The interactions monitored occurred between the residues of the WW1 and the WW2 domain, with a cut-off distance of 4Å. Donors and acceptors residues are summarized in the table where for each interaction an average value of the respective occurrence, calculated along the trajectory, is reported.
